# Husbandry and breeding practices of alpaca farmers in Ecuador and Peru: an exploratory study

**DOI:** 10.1007/s11250-025-04767-z

**Published:** 2025-12-09

**Authors:** Jana Marešová, Tersia Kokošková, Tamara Fedorova

**Affiliations:** Department of Animal Science and Food Processing, Faculty of Tropical AgriSciences, Czech University of Life Sciences Prague, Kamýcká 129, Praha - Suchdol, 165 00 Czech Republic

**Keywords:** Alpaca, Calving season, Farmers’ gender, Husbandry problems, Reproductive management

## Abstract

South American camelids (SACs) play an important socioeconomic role in the Andean regions. This study aimed to investigate the differences in alpaca farming and husbandry practices between Ecuador and Peru, and to analyse the effects of the farmers’ gender and the length of SACs farming experience thereon. A total of 102 alpaca (*Vicugna pacos*) farmers completed a questionnaire. The mean SACs farming experience was significantly lower in Ecuador than in Peru, and more experienced farmers kept significantly larger alpaca herds. Women were more often involved in alpaca farming than men in Peru. The main purpose of alpaca farming was for fibre and meat production. In Ecuador, 68.2% of the farmers kept the breeding males and females together during the whole year, while in Peru, 69.0% of farmers kept them separately and managed their breeding period. Significant calving seasonality was observed in both countries, with the peak of calving season in February. Farmers from both countries indicated that the most common problems included internal and external parasites, as well as a lack of pasture available. These are areas that require further attention from researchers and governments to enhance the sustainability of alpaca production. Additionally, understanding possible barriers for women in Ecuador regarding involvement in alpaca farming could contribute towards future development efforts.

**Supplementary Information:**

The online version contains supplementary material available at 10.1007/s11250-025-04767-z.

## Introduction

South American camelid (SAC) farming has formed an integral part of the traditional Andean way of life (Markemann and Valle Zárate 2010). Based on FAO reports (2023), there are 5.6 million SACs in Peru, and the number of alpacas in the country has increased between 2007 to 2022 from 3.69 million to 4.53 million (MIDAGRI 2023). In Ecuador, the population of SACs was almost extinct in the past (Germana Cavero et al. 2016). FAO (2023) currently provides no data for the SACs population in Ecuador, but the number of alpacas is estimated to be between 3.8 and 6.7 thousand (Germana Cavero et al. 2016; Vilá and Arzamendia 2022).

SACs have many roles in the Andean society and contribute strongly to food security and the livelihoods of farmers in these regions (Vilá and Arzamendia 2022; Miranda-de la Lama and Villarroel 2023). Most alpacas are kept in small farms by indigenous rural farmers and communities. Women are important contributors for the local economy and alpaca production brings new opportunities for them (Germana Cavero et al. 2016). Fiber production and alpaca fibre processing have a long tradition in Andean countries (Gutierrez et al. 2018), and the meat of alpacas and llamas is often the main source of protein for local people (Arzamendia et al. 2021). Thus, alpacas are important pastoral animals for local people in Peru and Ecuador but research interest and scientific knowledge about camelids in these regions is still insufficient to address current challenges (Kishore et al. 2024), like climate change, outmigration, and social changes (Turin and Mendiola 2024). Moreover, studies focusing on SACs in Ecuador are scarce (Germana Cavero et al. 2016). This study aimed to characterize the husbandry and breeding practices of farmers raising alpacas in Ecuador and Peru, to investigate the differences between these countries, and consider the effects of the farmers’ gender and the length of SACs farming experience on the practices utilised.

## Materials and methods

**Fig. 1:**
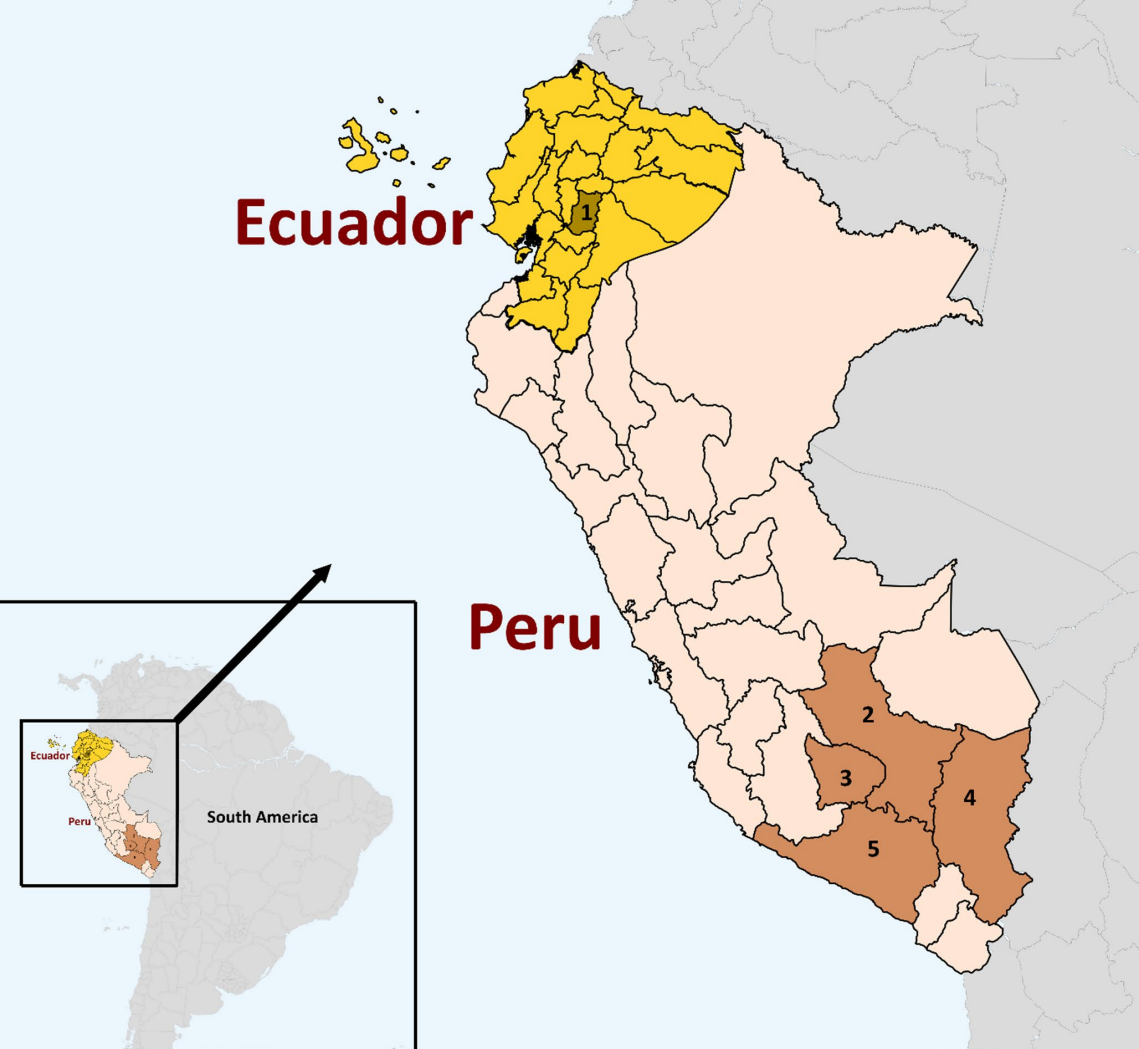
Ecuadorian and Peruvian regions (and number of interviews) included in the questionnaire study assessing husbandry practices of alpacas: 1 – Chimborazo (*n* = 44); 2 – Cusco (*n* = 8); 3 – Apurímac (*n* = 1); 4 – Puno (*n* = 15); 5 – Arequipa (*n* = 34)

Data about alpaca husbandry practices utilised in Ecuador and Peru was collected through self-administered questionnaires (*n* = 11) and face-to-face interviews (*n* = 91) from 102 selected alpaca farmers (Fig. 1). The data collection was carried out at alpaca breeding workshops for farmers and fibre producers or during animal auctions from April to July 2021 and April to September 2022. Participation in the interview was voluntary, and the farmers were informed about the purpose of the survey.

**Fig. 2:**
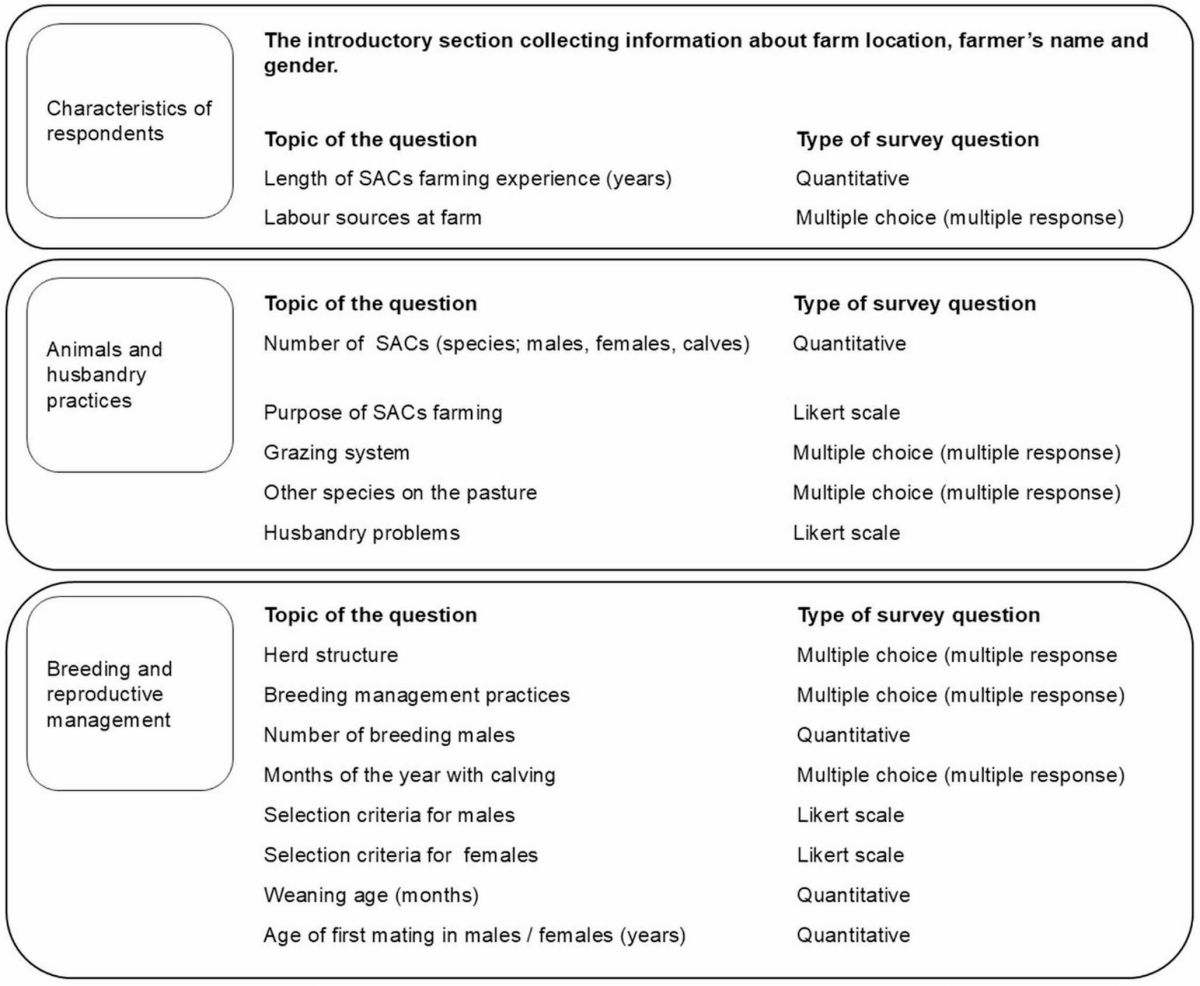
Summary of the questionnaire structure and types of survey questions used

The semi-structured questionnaire, in Spanish, was distributed in a paper-printed form. An English version is provided in Supplementary Material 1. The questionnaire, with 15 questions, focused on three areas which are summarized in Fig. 2. All personal data were anonymised for data handling and analyses.

The multiple-choice questions offered the respondents the option to select one or more answers from a preset list of options. The quantitative questions enabled respondents to provide numeric answers. Finally, the questions based on a 3-point Likert scale ranged from 1 = not important, 2 = secondary important, to 3 = priority/very important. Each question utilising the Likert scale contained seven to nine items which were scored independently. The selection criteria for breeding animals were chosen and adapted according to Markemann and Valle Zárate (2010). Respondents always had the opportunity to add their own answers, either by selecting ‘other’ and providing their own response, or by specifying additional selection criteria, but since this option was rarely used, these responses were not included in the data analysis and most results.

The statistical analysis was performed in Statistica version 13 (TIBCO Software Inc. 2017). Frequency tables and Pearson Chi-square test were used for the comparison of the occurrence of categorical variables like country, gender of farmer, labour sources, length of SACs farming experience categorised to three groups (1 to 10 years; 11 to 30 years; 31 + years), grazing practices, and managed/non-managed reproduction. Data from the 3-point Likert scales were treated like ordinal data where the median was calculated and displayed in spider diagrams. Since the quantitative data did not show a normal distribution (Kolmogorov-Smirnov test, *p* < 0.01), non-parametric Mann-Whitney U or Kruskal-Wallis tests were used. To explore the relationship between length of farming experience and herd size or age of weaning, Spearman’s rank correlation coefficient was determined. The level of significance used throughout the analyses was *p* < 0.05.

The circular statistics for testing the seasonality of calving season in Peru and Ecuador were performed in Oriana, version 4.02 (Kovach Computing Services 2013). Herds were classified as managed or unmanaged in terms of mating. Managed herds were herds where males were introduced to herd just for limited time period for mating. In non-managed herds, males were present with females all year. Rayleigh Test with a significance level *p* < 0.01 was used for these analyses.

## Results

### Characteristics of alpaca farmers and their herds

Characteristics of respondents are shown in Table 1. The owners of the animals themselves were primarily the only person involved in the farming activities (Table 1), especially for Peruvian farmers (*p* < 0.0001) and women farmers (63.3% for women vs. 24.5% for men; *p* < 0.0001).

**Table 1:** Characteristics of respondents and their distribution based on country, gender, length of sacs farming experience and labour sources

Characteristics	Total		Ecuador		Peru	
	Number	Percentage	Number	Percentage	Number	Percentage
**Overall**	102	100.0%	44	43.1%	58	56.9%
**Gender of farmer**						
Women	49	48.0%	7	15.9%	42	72.4%
Men	53	52.0%	37	84.1%	16	27.6%
**Length of SACs farming experience**						
1 to 10 years	44	43.1%	34	77.3%	10	17.3%
11 to 30 years	32	31.4%	10	22.7%	22	37.9%
31 + years	26	25.5%	0	0.0%	26	44.8%
**Labor sources at farms**						
Owner only	44	43.1%	5	11.4%	39	67.2%
All family members	35	34.3%	17	38.6%	18	31.0%
All adult family members	1	1.0%	0	0.0%	1	1.7%
Owner and paid workers	17	16.7%	15	34.1%	2	3.4%
Paid workers only	13	12.7%	10	22.7%	3	5.2%

Mean ± SEM SACs farming experience was significantly (*p* < 0.001) lower in Ecuador than in Peru (7.2 ± 1.20 vs. 31.3 ± 2.59, respectively). Women farmers had significantly (*p* < 0.001) longer experience than men (28.9 ± 3.07 vs. 13.5 ± 2.01, respectively).

Peruvian herds were significantly (*p* < 0.001) larger than Ecuadorian (Table 2). More experienced farmers kept significantly larger herds (*p* < 0.05, ρ = 0.57). Eleven farmers also kept llamas, and all these farmers had a minimum of 15 years of experience.

**Table 2:** Characteristics of herds according to country

Country	Mean ± SEM herd size	Median herd size	Minimum herd size	Maximum herd size	Mean ± SEM number of males (M)	Mean ± SEM number of females (F)	Mean ± SEM number of crias until weaning	Mean ± SEM sex ratio (M: F)
Ecuador	52.6 ± 8.70	27	6	250	11.70 ± 2.61	30.55 ± 4.40	10.36 ± 2.17	1:5.19 ± 0.53
Peru	225.8 ± 30.98	143	34	1100	33.00 ± 7.32	134.83 ± 18.25	58.00 ± 8.67	1:8.64 ± 2.18

**Fig. 3:**
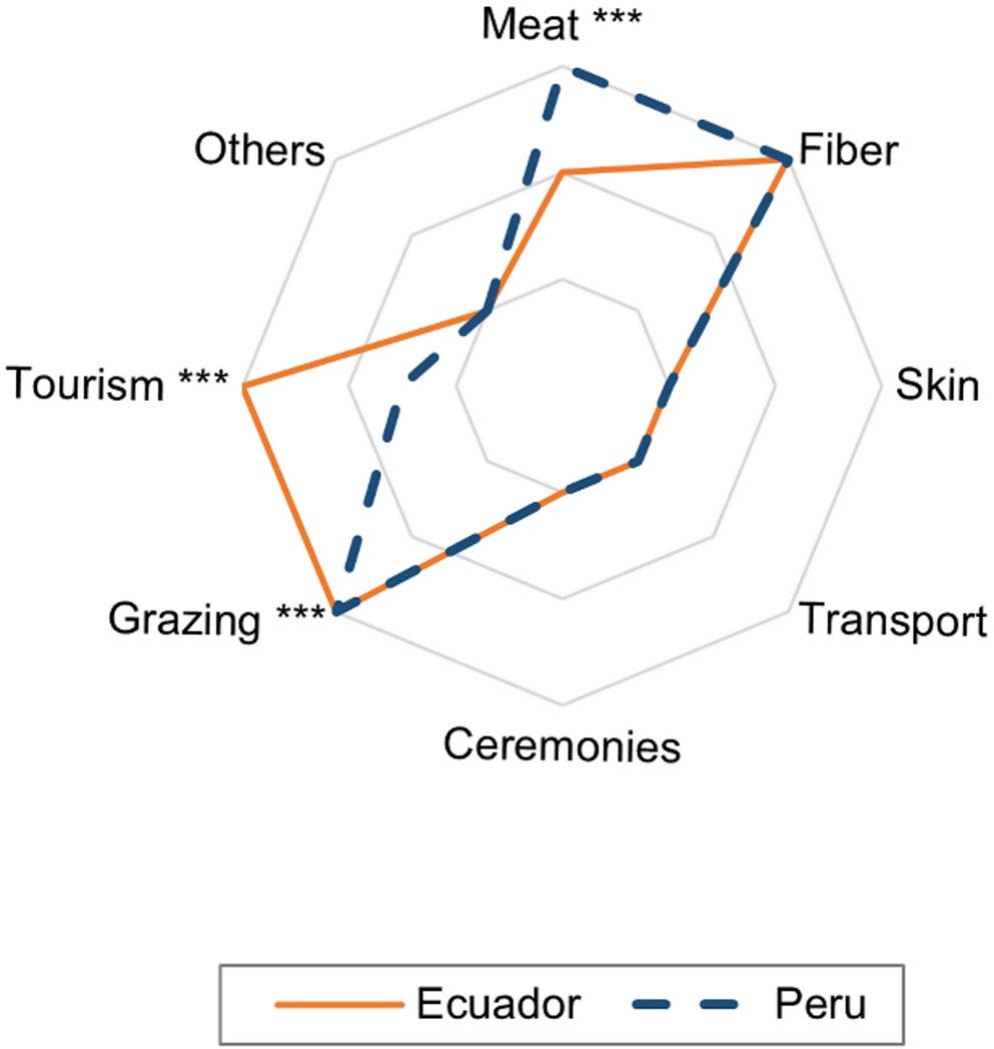
Median scores of importance of alpaca farming purposes in Ecuador and Peru (3: very important – primary purpose, 2: important – secondary purpose, 1: – not important). “Grazing” refers to the functional role of alpacas in pasture use and land maintenance. Asterisks mark significant differences between countries (*p < 0.05, **p < 0.01, *** *p* < 0.001)

96.6% and 94.8% of Peruvian farmers scored fibre and meat production as the most important purposes of their alpaca farming activities (see details in Fig. 3), whilst in Ecuadorian farmers, it was scored as 86.4 and 75.0%, respectively.

Extensive grazing, i.e. grazing on large natural pastures without any management, was the most common form of grazing/feeding used (77.5%). 97.1% of farmers did not provide any supplementary feeding to their alpacas. 72.6% farmers used their pasture exclusively for SACs. If other species of livestock were kept with alpacas, these were sheep (27.5%), cattle (10,8%), horses (2,9%), and donkeys (1.0%). No significant difference (*p* ˃ 0.05) was observed between countries in the above-mentioned practices. Ecuadorian farmers used herdsmen significantly more frequently than Peruvian farmers (47.7 vs. 15.5%, *p* ˃ 0.001). Internal and external parasites together with lack of pasture were noted by the majority of farmers as the most common serious problems for their farms.

### Breeding and reproductive management

In Ecuador, 68.2% of the farmers (*n* = 30) kept males and females together during the whole year, while in Peru, 69.0% (*n* = 40) of farmers kept males and females separately, and controlled mating. The majority of farmers in Ecuador and Peru (75.0% vs. 100.0%, respectively) had more than 1 breeding male in their herds of females (*p* = 0.001). The mean number of breeding males at one farm significantly differed between Ecuador and Peru (2.59 and 8.17, respectively; *p* < 0.001).

**Fig. 4:**
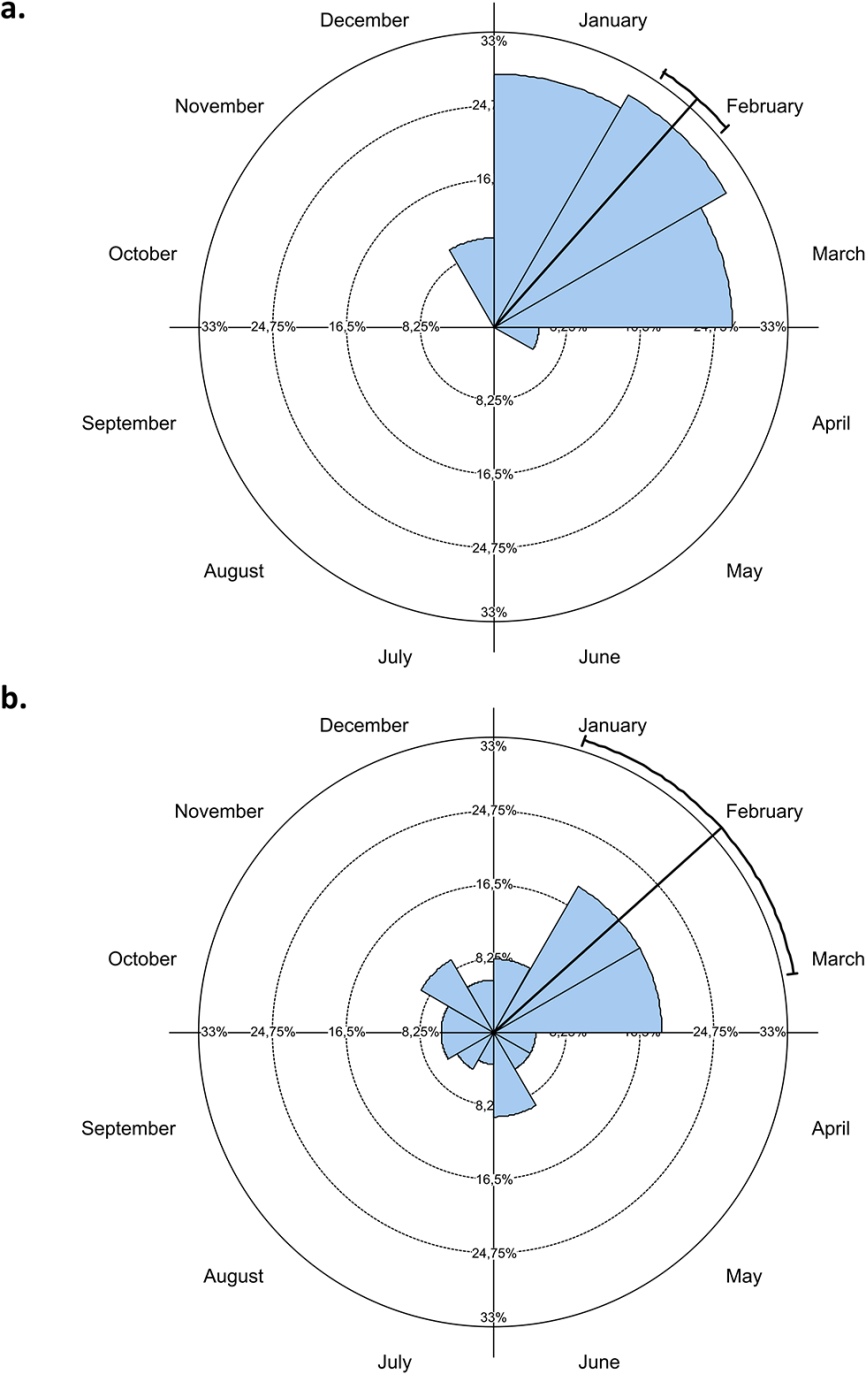
Distribution of cria births during the year (%) in non-managed alpaca herds in Peru (**a**) and Ecuador (**b**). Mean and confidence interval 95% are plotted

No significant difference (*p* ˃ 0.05) in calving season between managed (with reproductive management) and non-managed herds was found. February and March were common months of calving (80.4% and 77.5% of respondents recorded these months, respectively). In non-managed herds, significant seasonality was observed in both countries, with the peak of the calving season in February (*p* < 0.01). However calving seasons differed between Peru and Ecuador (*p* < 0.0001). As shown in Fig. 4, circular variance was higher in Ecuador than in Peru (0.726 vs. 0.137, respectively).

**Fig. 5:**
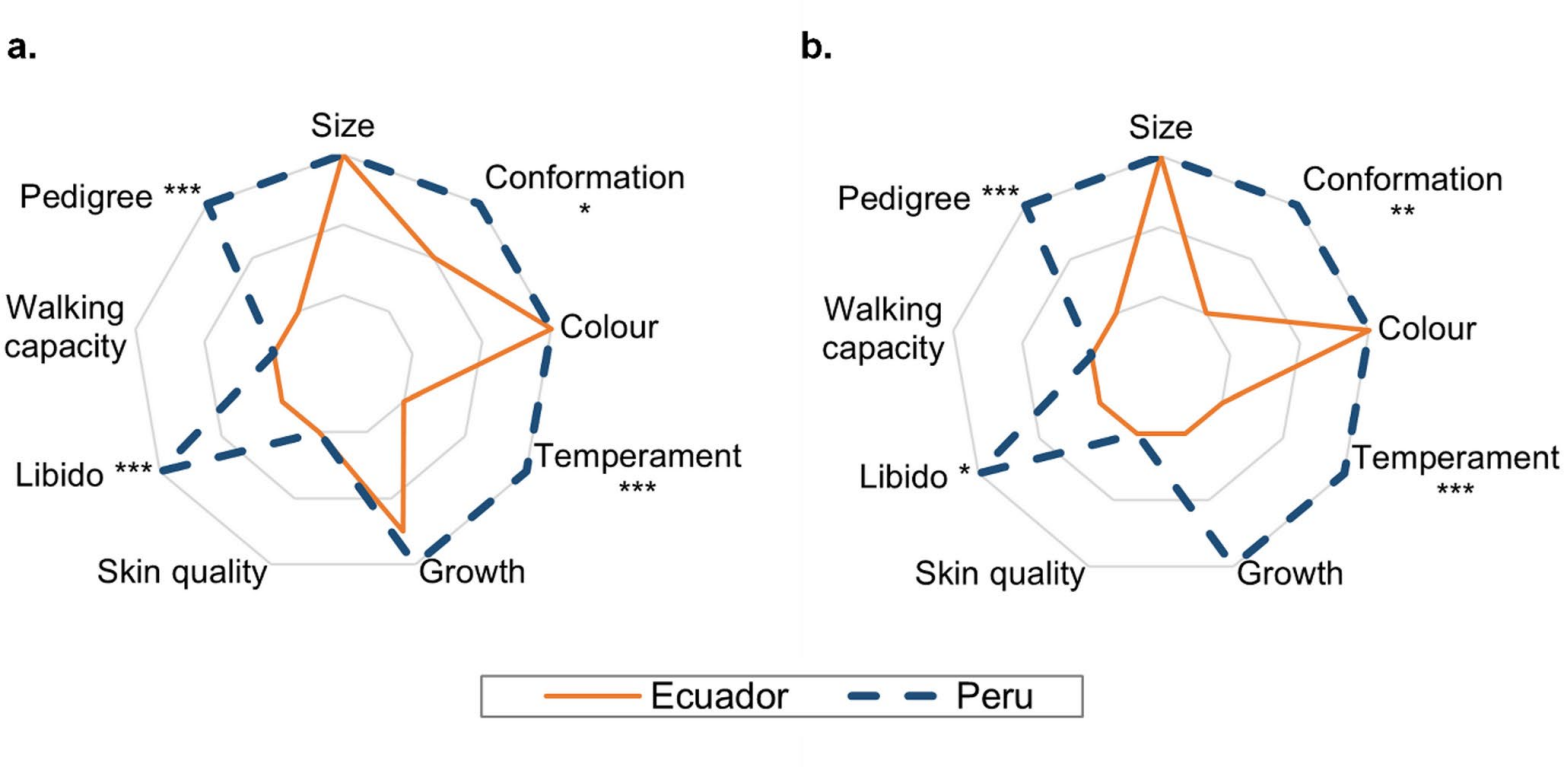
Median values of highly important (3) to non-important (1) alpaca male (**a**) and female (**b**) selection criteria according to the opinion of alpaca farmers in Ecuador and Peru. Asterisks mark significant differences between countries (*p < 0.05, **p < 0.01, *** *p* < 0.001)

Median scores for selection criteria for males and females in Ecuador and Peru are shown in Fig. 5. In addition to the preset selection criteria, 3 Peruvian farmers also mentioned wool quality. No significant differences in selection criteria were found between women and men farmers, just that women marked the pedigree of female animals as a primary selection criterion significantly more often than men farmers (57.1 vs. 28.3%, respectively; *p* = 0.012).

The reported reproductive parameters are presented in Table 3. The majority of farmers in Ecuador and Peru reported that they practice controlled weaning of crias (77.3% and 77.6%, respectively). However, only 35.3% of farmers in Ecuador and 13.3% of farmers in Peru separated crias from the herds after weaning. More experienced farmers tended to wean crias at an early age (*p* < 0.05, ρ = -0.43).

**Table 3:** Reproductive parameters (± SEM) in Ecuador and Peru

	Overall	Ecuador	Peru	*p*-value
Age at first mating in females (years)	2.1 ± 0.05	2.1 ± 0.08	2.2 ± 0.07	0.358
Age at first mating in males (years)	2.8 ± 0.06	2.6 ± 0.09	2.9 ± 0.08	0.034
Weaning age of crias (months)	8.9 ± 0.31	9.2 ± 0.66	8.6 ± 0.2	0.100

## Discussion

In the present study, Peruvian farmers had more farming experience and kept larger herds than farmers in Ecuador. Vilá and Arzamendia (2022) reported that llama and alpaca husbandry has a long tradition in Peru. In Ecuador, SAC farming was historically not so widespread (Germana Cavero et al. 2016). But in recent years, Ecuador focuses on strengthening capacities to develop the SAC industry in the country (FAO 2024).

In the present study, there were more women farmers that responded in Peru, and who also had more experience with alpaca farming than men. The importance of women participating in SACs farming was also reported by Calle (2023), as women are the ones who keep the alpaca farming tradition and transfer the knowledge to next generations. Conversely, in the present study, men were more involved in alpaca farming in Ecuador, which is likely due to the lower historical distribution of SAC farming (Germana Cavero et al. 2016). Alpaca farming is often practiced as a family business, but involvement of paid workers is common in Ecuador. In Peru, alpaca farming is often a communal activity, with families cooperating in its management (Sanchez 2004).

Alpaca farming has many purposes for the Andean farmers, such as fibre, meat, skin and others (Fig. 3), (Reyna 2006; Kishore et al. 2024). Fibre production is typical for alpacas (Gutierrez et al. 2018). Reported mixed grazing systems with other species can provide better economic security (Sanchez 2004; Radolf et al. 2022) but can also promote the transmission of viruses and parasites between the species, and it can influence fertility, birth mortality rates, and cause other reproductive problems (Cruz et al. 2020; Prince et al. 2016). Additionally, mixed herds of alpacas and llamas can lead to hybridisation (Markemann and Valle Zárate 2010).

Males were used for breeding from 2 to 3 years of age and crias were usually weaned at the age of 6 to 8 months as recommended (Reyna 2006; Ayala-Vargas 2018; Zárate et al. 2021). However, the mean age at the first breeding of alpaca females was much later than recommended by Sumar (1996). The lack of pasture could play a role as it was reported as a serious problem by most Peruvian farmers in the present study. In the rainy season, pasture conditions improve for SACs in the Peruvian Andes (Prince 2016; Cruz et al. 2020), while in Ecuador, rain occurs mostly all-year-round. This is also probably the reason why the breeding season in Peru occurs predominantly from January to March (Garnica et al. 1993; Sumar 1996) whilst it is more varied in Ecuador. Controlled mating practiced more frequently in Peru, might be attributed to the longer farming experience of the Peruvian farmers or other factors like differences in husbandry approaches, farmer trainings or tradition in both countries. Controlled mating may have a positive effect on alpaca male libido and fertility (Urquieta et al. 2005). Most farmers reported controlled weaning; however, just few of them separated the crias from the mothers after the weaning. So the effectivity of weaning practices should be verified.

There is always an inherent risk in using questionnaires with predefined responses. For example, the use of predefined traits originally developed for llamas (Markemann and Valle Zárate 2010) may have introduced a bias, as farmers might have conformed to the given options rather than expressing their usual selection criteria for alpacas, even though they had the opportunity to include their own criteria. This opportunity was used only by 3 respondents from Peru. This limitation could affect the accuracy of the results of selection criteria.

In conclusion, this preliminary study brings unique data from Ecuador and Peru about alpaca farming and husbandry practices. Although this survey brought limited data which cannot be applicable for the broader population due to the small number of respondents, several similarities and differences in husbandry and breeding practises can be seen between these two countries. Farmers from both countries face the same problems, like internal and external parasites and lack of pasture for their animals, which should be an area of focus for local organisations. Research of alternative feed sources for SACs and proper management of pastures, could help the farmers to mitigate these problems and to face issues linked with climate change challenges. The role of alpacas in both countries is important and since the study represents a small number of farmers, the further investigation of SACs farmers’ needs and limitations is necessary to plan proper support.

## Supplementary Information

Below is the link to the electronic supplementary material.

Supplementary Material 1

## Data Availability

The datasets generated during and/or analysed during the current study are available from the corresponding author on reasonable request.
