# Supplementary material for: Husbandry and breeding practices of alpaca farmers in Ecuador and Peru: an exploratory study
Source: Trop Anim Health Prod. 2025 Dec 9;57(9):535. doi: 10.1007/s11250-025-04767-z (PMC12689803; doi:10.1007/s11250-025-04767-z)
Supplement: Supplementary file 1 — Supplementary Material 1 [file 11250_2025_4767_MOESM1_ESM.docx]

**Husbandry and breeding practices of alpaca farmers in Ecuador and Peru: an exploratory study**

Tropical Animal Health and Production

Jana Marešová, Tersia Kokošková, Tamara Fedorova*

Department of Animal Science and Food Processing, Faculty of Tropical AgriSciences, Czech University of Life Sciences Prague, Kamýcká 129, 165 00 Praha - Suchdol, Czech Republic

*fedorova@ftz.czu.cz

**Questionnaire**

**Information about the farm and respondent**

Date:

Location:

Farm:

Respondent name & gender:

1. **What is the purpose of your alpaca breeding?**

|  | Primary | Minor | Unimportant |
| --- | --- | --- | --- |
| Meat |  |  |  |
| Fibre |  |  |  |
| Skin |  |  |  |
| Transport |  |  |  |
| Ceremonies |  |  |  |
| Grazing |  |  |  |
| Tourism |  |  |  |
| Other: |  |  |  |

1. **How long do you breed alpacas?**

__________________________

1. **How many South American camelids do you breed?**

|  | Llamas | Alpacas | Others |
| --- | --- | --- | --- |
| Males |  |  |  |
| Females |  |  |  |
| Calves (until weaning) |  |  |  |

1. **How many people are involved in your alpaca breeding?**

Owner himself only

All family members

All adult family members

Owner and paid workers

Only paid (hired) workers

Others __________________________

1. **What is your type of grazing?**

Free extensive pasture

Pasture with shepherd

Mixed grazing with other livestock

Grazing and supplementary feeding

Others _______________________________________________

1. **If you apply mixed grazing, what are the others animal species?**

Cattle

Sheep

Horses

Donkeys

Others _______________________________________________

1. **What is your herd organization?**

Males and females separately

Males and females together

One female per flock of females

Young separated after weaning

The young remain with mother even after weaning

Variable by season

Others _______________________________________________

1. **What are the months of births?**

January

February

March

April

May

June

July

August

September

October

November

December

1. **What breeding management methods do you use?**

None – all animals in one herd all year around

Controlled admission - selected males with females on mating season

Males and females are selected for mating

Controlled weaning – all young leave the herd at the age of …….

Young males leave the herd at age …….. females remain in the herd

Note

1. **At what age are animals included in breeding?**

Males ________ months

Females ________ months

1. **What are you selection criteria for the males?**

| Males | Priority | Secondary | Not important |
| --- | --- | --- | --- |
| Size |  |  |  |
| Conformation |  |  |  |
| Colour |  |  |  |
| Temperament |  |  |  |
| Growth rate |  |  |  |
| Skin quality |  |  |  |
| Libido |  |  |  |
| Long distance walking capacity |  |  |  |
| Pedigree |  |  |  |
| Others | | | |

1. **What are you selection criteria for the females?**

| Females | Priority | Secondary | Not important |
| --- | --- | --- | --- |
| Size |  |  |  |
| Conformation |  |  |  |
| Colour |  |  |  |
| Temperament |  |  |  |
| Growth rate |  |  |  |
| Skin quality |  |  |  |
| Libido |  |  |  |
| Long distance walking capacity |  |  |  |
| Pedigree |  |  |  |
| Others | | | |

1. **How many males do you use for breeding?**

_______________

1. **At what age are the young ones weaned?**

_______________

1. **What are the main problems in your alpaca breeding?**

|  | Serious problem | Slight problem | Non problematic |
| --- | --- | --- | --- |
| Lack of pastures |  |  |  |
| Congenital malformations of calves |  |  |  |
| Internal parasitic diseases |  |  |  |
| External parasitic diseases |  |  |  |
| Infectious diseases |  |  |  |
| Fertility problems |  |  |  |
| Technical problems or handling problems |  |  |  |
| Administrative problems |  |  |  |
| Economic problems |  |  |  |
